# Supplementary material for: Comparative Transcriptomic Analysis Reveals Adaptation Mechanisms of Bean Bug Riptortus pedestris to Different Food Resources
Source: Insects. 2023 Aug 31;14(9):739. doi: 10.3390/insects14090739 (PMC10531862; doi:10.3390/insects14090739)
Supplement: Supplementary file 1 [file insects-14-00739-s001.zip › insects-2497244-supplementary/insects-2497244-supplementary/Table S1.pdf]

Table S1 Information of qRT-PCR primers

Primer name	Forward primer	Reverse primer
qRCR-RpCSL	TCTTGCTGGGCTTTCTC	TATGGGTATGACTCTTCTGTG
qRCR-RpCYP6	TACGGTCGAGGTTATCTG	GGGCTGGTTATCCTTACT
qRCR-RpActin	CTCCTGAGTCAAGCACAATA	GCATCACACCTTCTACAATG
qRCR-RpCBP6	AGGTGGTTCTATTGACTGG	TCACGCTTTGGATGCTG
qPCR-RpVg	AAACACTGCTCACCCTAA	TTGGTCCTCATCAGTTGTC
qPCR-Rp32478	TCTGCTTCCTCGCTGTT	ATGGCGTTGGTGATGTT
qPCR-RpALP	CCAGGCAAACATCAAGC	ATCCCAGAGCAACCACC
qPCR-Rp51213	CAAGTGCTTGGCCTCTAC	AGGTGCGGGTGAATCTG
qPCR-Rp52328	GAGTGACGGAAGGGTGG	ACGGAGCAGTGTATGTGAG
qPCR-Rp10883	ACAACAGTCCAGCCAGTA	GACCACTTCCCTCATCT
qPCR-Rp12557	ACAACAATACCCGAAAG	CTCATCATCAGGCAGTC
qPCR-Rp12609	TATGAGAACCGGATGTG	AAGTAGCCATTGACCTG
qPCR-Rp40087	TTGGAGTAATCGGAGGG	GCACGGATAGAAACAGC
